# Impact of Environmental Exposures on Human Breast Milk Lipidome in Future Immune-Mediated Diseases

**DOI:** 10.1021/acs.est.3c06269

**Published:** 2024-01-24

**Authors:** Tuulia Hyötyläinen, Tannaz Ghaffarzadegan, Bagavathy Shanmugam Karthikeyan, Eric Triplett, Matej Orešič, Johnny Ludvigsson

**Affiliations:** † School of Science and Technology, Örebro University, Örebro SE-702 81, Sweden; ‡ Department of Microbiology and Cell Science, Institute of Food and Agricultural Sciences University of Florida, Gainesville, Florida 32611-0700, United States; § School of Medical Sciences, Faculty of Medicine and Health, Örebro University, Örebro SE-702 81, Sweden; ∥ Turku Bioscience Centre, University of Turku and Åbo Akademi University, Turku FI-20520, Finland; ⊥ Crown Princess Victoria’s Children’s Hospital and Division of Pediatrics, Department of Biomedical and Clinical Sciences, Linköping University, Linköping SE 58185, Sweden

**Keywords:** human breast milk, perfluorinated alkyl substances, autoimmune diseases, lipidomics, maternal factors

## Abstract

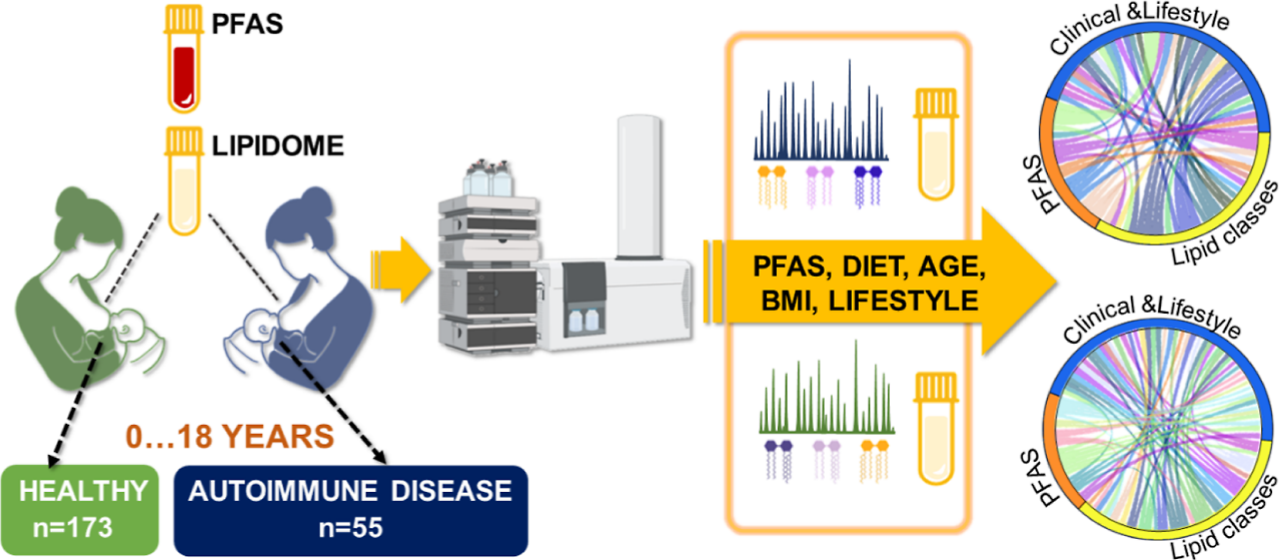

The composition of human breast milk (HBM) exhibits significant variability both between individuals and within the same individual. While environmental factors are believed to play a role in this variation, their influence on breast milk composition remains inadequately understood. Herein, we investigate the impact of environmental factors on HBM lipid composition in a general population cohort. The study included mothers (All Babies In Southeast Sweden study) whose children later progressed to one or more immune-mediated diseases later in life: type 1 diabetes (*n* = 9), celiac disease (*n* = 24), juvenile idiopathic arthritis (*n* = 9), inflammatory bowel disease (*n* = 7), hypothyroidism (*n* = 6), and matched controls (*n* = 173). Lipidome of HBM was characterized by liquid chromatography combined with high-resolution mass spectrometry. We observed that maternal age, body mass index, diet, and exposure to perfluorinated alkyl substances (PFASs) had a marked impact on breast milk lipidome, with larger changes observed in the milk of those mothers whose children later developed autoimmune diseases. We also observed differences in breast milk lipid composition in those mothers whose offspring later developed autoimmune diseases. Our study suggests that breast milk lipid composition is modified by a complex interaction between genetic and environmental factors, and, importantly, this impact was significantly more pronounced in those mothers whose offspring later developed autoimmune/inflammatory diseases. Our findings also suggest that merely assessing PFAS concentration may not capture the full extent of the impact of chemical exposures; thus, the more comprehensive exposome approach is essential for accurately assessing the impact of PFAS exposure on HBM and, consequently, on the health outcomes of the offspring.

## Abstract 2

Maternal factors and exposure to perfluorinated alkyl substances impact breast milk lipid composition, particularly in mothers with children prone to autoimmune diseases.

## Introduction

Human breast milk (HBM) is a highly complex biological fluid encompassing a variety of essential macronutrients, including lipids, proteins, carbohydrates, and vitamins, that are crucial for the healthy growth and development of infants. These macronutrients are emulsified in an aqueous milk matrix, and their composition is precisely balanced to cater the distinct nutritional requirements of the developing child.^1^ The importance of early life nutrition in shaping long-term health outcomes cannot be overstated. Breastfed infants have been shown to experience a range of benefits, including reduced risk of infections, improved gut and intestinal development, and better regulation of body weight, even long after they have stopped breastfeeding.^1−3^ Moreover, there is evidence to suggest that breastfeeding may help protect against type 1 diabetes (T1D) and other autoimmune diseases.^4,5^ Breastfeeding also confers important health benefits to nursing mothers. Several studies have shown that women who breastfeed have a decreased risk of ovarian and breast cancers.^6−8^

Lipids are a critical component of breast milk, constituting the third most abundant constituent. These lipids not only provide infants with a significant source of energy (approximately 50% of their total energy from HBM) but also have a crucial role in the development and health of the infants.^9,10^ The lipids present in breast milk include triacylglycerols (TGs), glycerophospholipids, sphingolipids, sterols, and glycolipids. The large majority of the total lipid content in HBM (>98%) is made by TGs which provide with ca. half of the total dietary energy demand of breastfed infants.^11,12^ The composition of breast milk is dynamic, with variations occurring during feeding, diurnally, over lactation, and influenced by diet, genetic background, lifestyle, and body mass index (BMI) of the mother.^13^ The chemical composition of HBM also undergoes alterations from colostrum to later stages of lactation, with the lipid composition being the most highly variable macronutrient and with fat concentration and composition changing with lactation stage.^14−16^ Variation in the fatty acid (FA) composition of breast milk lipids has been linked with infant growth, neurocognitive development and function, inflammatory regulation and infection risk, as well as risk of metabolic and cardiovascular diseases later in life.^17^ The FA composition has also been shown to be influenced by both long- and short-term maternal dietary habits.^18^ Multiple other maternal parameters may potentially have an impact on the FA composition of the HBM such as maternal BMI and genetic variants of FA desaturase gene.^18,19^ Recent studies have explored the potential impact of birth weight, gestational age, infant age/stage of lactation, and maternal factors, including lifestyle and sociodemographic factors, on the composition of breast milk. While there is existing knowledge regarding the effect of the maternal diet on breast milk composition, there is still much to be discovered about the influence of other maternal factors.^19−22^ Maternal exposure to environmental chemicals, primarily through her diet, may also impact breast milk composition.^23,24^

We have recently demonstrated in a pilot study that circulating polyfluoroalkyl substances (PFASs) were associated with the HBM lipid composition, with high exposure being associated with reduced nutritional quality of HBM, with reduction in total lipid content, reduced concentration of polyunsaturated FAs (PUFAs), such as lipids containing docosahexaenoic acid- and arachidonic acid, while lipid containing saturated FAs were increased with higher PFAS exposure.^23^ PFASs are industrial chemicals that are used in a wide range of industrial and consumer applications, and their persistence in the environment has led to their widespread distribution in water, soil, wildlife, and humans.^25−27^ Epidemiological studies indicate a potential inverse relationship between maternal PFAS levels and duration of breastfeeding.^28−31^ Studies in animal models have shown that exposure to PFAS during gestation can lead to impaired mammary differentiation and stunted mammary epithelial development in offspring.^32^ Together, the current data suggest that accumulated PFAS exposure may disrupt lactation and alter the composition of breast milk.

Our pilot study included infants at increased genetic risk for T1D and thus was not representative of the general population. Herein, in a general population study, we investigated the impact of various environmental factors on HBM lipid composition, including dietary factors, smoking, and PFAS serum level at delivery. We investigated the overall changes as well as compared these changes with changes in a subgroup of mothers whose children later developed specific immune-mediated diseases (T1D, celiac disease [CD], juvenile idiopathic arthritis [JIA], irritable bowel disease [IBD], and hypothyroidism [HT]).

## Materials and Methods

### ABIS Cohort Study

All Babies In Southeast Sweden (ABIS) is a general population prospective birth cohort designed to identify environmental and genetic factors associated with immune-mediated diseases.^33^ The samples used in this study derived from the ABIS cohort comprise children born in Southeastern Sweden (specifically, Östergötland, Småland, Blekinge, and Öland) between October 1, 1997, and October 1, 1999. From the umbilical cord, the midwifes collected 1–6 mL of EDTA blood and also stored ca. 1 mL of serum, which was rapidly frozen in −20 °C and then transported to the biobank in Linköping where it was stored at −80 °C. The mothers answered comprehensive questionnaires at birth of their child and later at regular follow-ups, and biological samples were collected including breastmilk from the mothers and cord blood from the babies. Breast milk was collected 3 days postnatally at the obstetric clinics usually at 09–11 am and rapidly frozen at −20 °C and then transported to the biobank in Linköping. The mothers answered comprehensive questionnaires related to 22 questions on diet of the mothers during pregnancy (frequency data: daily, 3–5 times/week, 1–2 times/week, and more rarely) covering milk products, meat, sausages, fish, bread, potatoes, vegetables, fruits, mushrooms, sweets, and fast food. This information is further detailed and simplified in Table S1. We selected mother–infant dyads among children who later developed specific immune-mediated diseases, i.e., who later were diagnosed with either T1D, CD, IBD (Crohn’s disease and colitis ulcerosa), JIA, or HT, and controls who remained healthy during the follow-up, matched for period of birth and sex (Tables 1 and S1 and Figure S1). The Swedish National Diagnosis Registry provided the diagnoses. CD diagnosis was considered valid only when subjects received confirmation of the diagnosis after their initial assessment.

**Table 1:** Demographic Characteristics of the Study Cohort^a^

	all	controls	autoimmune	*p*
diagnosis (N)	228	173	55	
age (year)	29.5(7)	29(7)	30(6)	ns
BMI (kg/m^2^)	23(4.6)	23.1(4.3)	23(4.25)	ns
gestational age (weeks)	40(2)	40(2)	40(2.50)	ns
birth weight (g)	3550(702.5)	3550(670)	3540(805)	ns
delivery (vaginal/cesarean)	193/35	151/22	42/34	0.032
child sex (F/M)	104/124	81/92	23/32	ns

a Values shown as median interquartile range, unless noted otherwise.

The ABIS project has been approved by the Research Ethics Committees of the Faculty of Health Science at the University of Linköping, Linköping Sweden (refs 1997/96287 and 2003/03-092) and the Medical Faculty at the University of Lund, Lund, Sweden (DNR 99227 and DNR 99321). Participating families gave informed consent after oral and written information as well as the opportunity to watch a video of the study. ABIS to national registers was approved by the Research Ethics Committees of the Faculty of Health Sciences at Linköping University, Sweden, DNR 05-513 and 2018/380-32.

### Sample Preparation

For lipidomic analyses of HBM and serum, 30 μL of HBM or 10 μL of serum was extracted using a modified version of the previously published Folch procedure as described earlier.^23^ The samples were randomized before sample preparation and analysis. In short, 10 μL of 0.9% NaCl and 120 μL of CHCl_3_:MeOH (2:1, v/v) containing the internal standards (*c* = 2.5 μg/mL) was added to the sample. The standard solution contained the following compounds: 1,2-diheptadecanoyl-*sn*-glycero-3-phosphoethanolamine [PE(17:0/17:0)], *N*-heptadecanoyl-d-*erythro*-sphingosylphosphorylcholine [SM(d18:1/17:0)], *N*-heptadecanoyl-d-*erythro*-sphingosine [Cer(d18:1/17:0)], 1,2-diheptadecanoyl-*sn*-glycero-3-phosphocholine [PC(17:0/17:0)], 1-heptadecanoyl-2-hydroxy-*sn*-glycero-3-phosphocholine [LPC(17:0)], and 1-palmitoyl-d31-2-oleoyl-*sn*-glycero-3-phosphocholine [PC(16:0/d31/18:1)], were purchased from Avanti Polar Lipids, Inc. (Alabaster, AL, USA), and triheptadecanoylglycerol [TG(17:0/17:0/17:0)] was purchased from Larodan AB (Solna, Sweden). The samples were vortex-mixed and incubated on ice for 30 min, after which they were centrifuged (9400*g*, 3 min). 60 μL from the lower layer of each sample was then transferred to a glass vial with an insert, and 60 μL of CHCl_3_:MeOH (2:1, v/v) was added to each sample. The samples were stored at −80 °C until analysis.

For PFAS analyses, 40 μL of cord serum was extracted with 400 μL of cold MeOH/H_2_O containing labeled perfluorooctanoic acid (PFOA-^13^C8), perfluorononanoic acid (PFNA-^13^C5), perfluoroundecanoic acid (PFUndA-^13^C7), perfluorohexanesulfonic acid (PFHxS-^13^C3), and perfluorooctanesulfonic acid (PFOS-^13^C8), *c* = 0.2 μg/mL. Purchased from Wellington Laboratories (Guelph, Ontario, Canada). The tube was vortexed and ultrasonicated for 3 min, followed by centrifugation (10,000 rpm, 5 min). After centrifuging, 90 μL of the upper layer of the solution was collected and evaporated under nitrogen gas to the dryness. After drying, the sample was reconstituted into 60 μL of MeOH:H_2_O (70:30). The MeOH:H_2_O ratio was chosen to ensure efficient desolation of coextracted lipids. The samples were stored at −80 °C until analysis.

### Instrumental Analysis

The lipidomic analyses were done using an ultrahigh-performance liquid chromatography quadrupole time-of-flight mass spectrometry instrument (UHPLC-Q-TOF-MS) from Agilent Technologies (Santa Clara, CA, USA) as described previously.^23^ The liquid chromatography (LC) column was an ACQUITY UPLC BEH C18 column (2.1 × 100 mm, particle size 1.7 μm) by Waters (Milford, USA). Mobile phases are as follows: (A) 10 mM NH_4_Ac and 0.1% formic acid in water and (B) 10 mM NH_4_Ac and 0.1% formic acid in acetonitrile/isopropanol (1:1, v/v). Dual-jet stream electrospray [dual electrospray ionization (ESI)] ion source was used in the positive mode. The internal standard mixture was used for normalization, and lipid-class specific calibration was used for quantitation. Mass spectrometry (MS) data processing was performed using open source software MZmine 2.52.^34^ Identification of lipids was done by using an in-house laboratory based on LC-MS/MS data on retention time and mass spectra.

The PFAS analyses were performed with UHPLC-Q-TOF-MS (Agilent Technologies, Santa Clara, CA, The United States of America) with an Acquity UPLC, BEH C18 (2.1 × 100 mm, 1.7 μm) (Waters, Milford, MA, USA) column set at 50 °C with a C18 precolumn (Waters, Wexford, Ireland). The mobile phases are as follows: (A) 2 mM NH_4_Ac in H_2_O/MeOH (70:30, v/v) and (B) 2 mM NH_4_Ac in MeOH. The samples were kept at 10 °C, and injection volume was 10 μL. The flow rate was 0.4 mL/min, and the gradient started with 95% A and 5% B with a change after 1.5 min to 70% A and 30% B, which followed a change after 4.5 min to 30% A and 70% B; the last change was after 7.5 min with 100% B until the end of run. Dual-jet stream electrospray (dual ESI) ion source was used and with the ion polarity set on the negative mode. The capillary voltage and the nozzle voltage were kept at 4500 and 1500 V, respectively. The N_2_ pressure was set on 21 psi, with the sheath gas flow as 11 L/min and temperature at 379 °C for the nebulizer. The data was acquired with MassHunter B.06.01 software (Agilent Technologies, Santa Clara, CA, USA). MS data processing was performed using open source software MZmine 2.52.^34^ The identification of lipids was done with a custom database, with identification levels 1 and 2, i.e., based on authentic standard compounds (level 1) and based on MS/MS identification (level 2) based on Metabolomics Standards Initiative.^35^

Quantification of lipids was done using a 7-point internal calibration curve (0.1–5 μg/mL) and for PFAS using a 8-point calibration curve (*c* = 0.1–120 ng/mL). For lipids, the following lipid-class specific authentic standards were used: using 1-hexadecyl-2-(9Z-octadecenoyl)-*sn*-glycero-3-phosphocholine [PC(16:0*e*/18:1(9Z))], 1-(1Z-octadecenyl)-2-(9Z-octadecenoyl)-*sn*-glycero-3-phosphocholine [PC(18:0*p*/18:1(9Z))], 1-stearoyl-2-hydroxy-*sn*-glycero-3-phosphocholine [LPC(18:0)], 1-oleoyl-2-hydroxy-*sn*-glycero-3-phosphocholine [LPC(18:1)], 1-palmitoyl-2-oleoyl-*sn*-glycero-3-phosphoethanolamine [PE(16:0/18:1)], 1-(1Z-octadecenyl)-2-docosahexaenoyl-*sn*-glycero-3-phosphocholine [PC(18:0*p*/22:6)], 1-stearoyl-2-linoleoyl-*sn*-glycerol [DG(18:0/18:2)], 1-(9Z-octadecenoyl)-*sn*-glycero-3-phosphoethanolamine [LPE(18:1)], *N*-(9Z-octadecenoyl)-sphinganine [Cer(d18:0/18:1(9Z))], and 1-hexadecyl-2-(9Z-octadecenoyl)-*sn*-glycero-3-phosphoethanolamine [PE(16:0/18:1)] from Avanti Polar Lipids, 1-palmitoyl-2-hydroxy-*sn*-glycero-3-phosphatidylcholine [LPC(16:0)], 1,2,3 trihexadecanoylglycerol [TG(16:0/16:0/16:0)], 1,2,3-trioctadecanoylglycerol [TG(18:0/18:0/18)], 3β-hydroxy-5-cholestene-3-stearate [ChoE(18:0)], and 3β-hydroxy-5-cholestene-3-linoleate [ChoE(18:2)] from Larodan were prepared to the following concentration levels: 100, 500, 1000, 1500, 2000, 2500, and 5000 ng/mL (in CHCl_3_:MeOH, 2:1, v/v) including 1250 ng/mL of each internal standard. The correlation coefficients were >0.98 for all lipid classes. For PFAS, quantitation was done by six-point calibration curves using native calibration standards (PFBuS, PFBA, PFDA, PFDoDA, PFDoDS, PFDS, PFHpA, PFHxA, PFHxS, PFECHS, PFNA, PFNS, PFOA, PFOS, PFOSA, PFPeA, PFPeS, PFTDA, PFTrDA, and PFUnDA) from Wellington Laboratories (Guelph, Ontario, Canada). The calibration curves included zero and had correlation coefficients >0.99 for all PFASs. The limit of detection (3 × noise) is between 0.08 and 0.02 ng/mL, and the limit of quantitation (10 × noise) is between 0.04 and 0.1 ng/mL for the PFASs.

### Quality Control

Quality control was accomplished both for lipidomics and PFAS analysis by including blanks, pure standard samples, extracted standard samples, pooled quality control samples, and control plasma samples (NIST SRM 1950, purchased from the National Institute of Standards and Technology (NIST) at the US Department of Commerce (Washington, DC, USA)). In lipidomic analyses, lipids that had >30% relative standard deviation (RSD) in the pooled QC samples (an equal aliquot of each sample pooled together) or that were present at high concentrations in the extracted blank samples (ratio between samples to blanks <5) were excluded from the data analyses. Relative standard deviations (% RSDs) for lipids in the pooled samples (*n* = 14) were on average 11.8% (Table S2, Supporting Information Excel, and datasheet 1). The lipid concentrations in NIST CRM 1950 were in agreement of the consensus values reported earlier.^36^ For PFAS, the RSD in the pooled serum samples (*n* = 15) was on average 15.6% (Supporting Information Excel, datasheet 2). The PFAS values for PFOA, PFOS, PFNA, and PFHxS showed a good agreement with the reference values in the NIST CRM 19650 serum, being on average 90.4% (50.1–109.9%). The results for PFASs are not adjusted with the recovery.

### Statistical Analysis

All statistical analyses were performed at both the level of individual lipid concentrations and the level of lipid classes. For lipid classes, individual lipid concentrations in each lipid class were first median-normalized and summed, and then subsequent data analysis considering each lipid class as a variable was performed. For regression analysis, the individual PFAS concentrations were used as exposure variable (Table 2). The data was log-transformed and autoscaled prior to the statistical analyses. A linear model with covariate adjustments were done using the limma method in MetaboAnalyst 5.0.^37,38^ Age, BMI, delivery type, and gestational age (in weeks) were considered as covariates (Table S1). Partial correlations were calculated after adjustment with maternal age and BMI, and the partial correlations showing significant (*p* < 0.05, FDR < 0.1) were visualized using a Chord plot.^39^

**Table 2:** PFAS Concentrations in the Cord Blood (ng/mL) in the Whole Cohort and in Mothers Whose Children Did Not Develop Autoimmune Diseases during the Follow-Up Period (18 Years) and in Mothers Whose Children Developed Autoimmune Diseases

maternal group		PFDA	PFHxS	PFOA	PFOA_Br	PFOS	PFOS_Br	PFTrDA
All	MEDIAN	0.08	0.11	0.57	2.20	1.71	3.02	0
	MIN	<LOQ	<LOQ	0.07	0.40	0.81	1.39	<LOQ
	MAX	0.13	23.65	3.04	6.96	16.85	33.21	0.04
CTRL	MED	0.08	0.06	0.67	2.66	1.90	3.47	0
	MIN	<LOQ	<LOQ	<LOQ	<LOQ	0.38	0.68	<LOQ
	MAX	0.20	66.59	4.59	13.06	40.74	74.17	0.04
CASES	MED	0.08	0.11	0.56	2.21	1.71	3.06	0.01
	MIN	<LOQ	<LOQ	0.07	0.40	0.81	1.39	<LOQ
	MAX	0.13	23.65	3.04	6.96	16.85	33.21	0.04

## Results and Discussion

### Maternal Breast Milk Lipidome and PFAS Levels

PFAS levels were measured in cord serum samples, reflecting the maternal exposure. Six PFASs were detected and quantified in >70% of the samples, namely, PFDA, PFHxS, two isomers of PFOA, and two isomers of PFOS (linear and branched) (Table 2). Both isomers of PFOA were significantly lower in the case group (*p* = 0.014 and 0.016 for linear and branched PFOA, respectively). Interestingly, for PFOS and PFOA, the branched chain isomers had a higher concentration than the linear isomer. This could be due to branched PFAS accumulating relatively more than the linear form as reported both in humans^40^ and in animals^41^ or due to either higher intake or lower elimination. In addition, branched isomers of PFOS and PFOA have been reported to have a higher *trans*-placental transfer efficiency than the linear isomers^42^ that could explain the higher levels of the branched isomers specifically in cord blood. In addition, most studies that have reported the quantitative results for branched chain isomers have been using a triple quadrupole system while using a linear isomer for quantitation; due to difference in fragment intensities, this approach tends to underestimate the concentration of the branched isomers, unlike with our method that is based on high-resolution MS. In general, the PFAS concentrations in the cord blood were similar to those reported in previous studies collected on similar time, although these studies have not been reporting the branched-chain isomers.^43−46^ The PFAS concentrations showed both positive and inverse associations with maternal parameters and lifestyle after adjustment with BMI (Figure S3). The branched-chain PFOS and linear PFOA showed positive association with maternal BMI, while age did not show any significant association. Maternal education level was inversely associated with branched-chain PFOS. Consumption of eggs was positively associated with linear PFOA and both isomers of PFOS and pork consumption with branched-chain PFOA. Use of antibiotics, hormones, and other medication also showed both positive and adverse associations with both isomers of PFOA and linear PFOS. Fish intake during pregnancy did not show significant associations with the PFAS. However, it should be noted that due to dietary guidelines during pregnancy, the reported fish intake may not reflect the long-term fish consumption.

**Figure 1:**
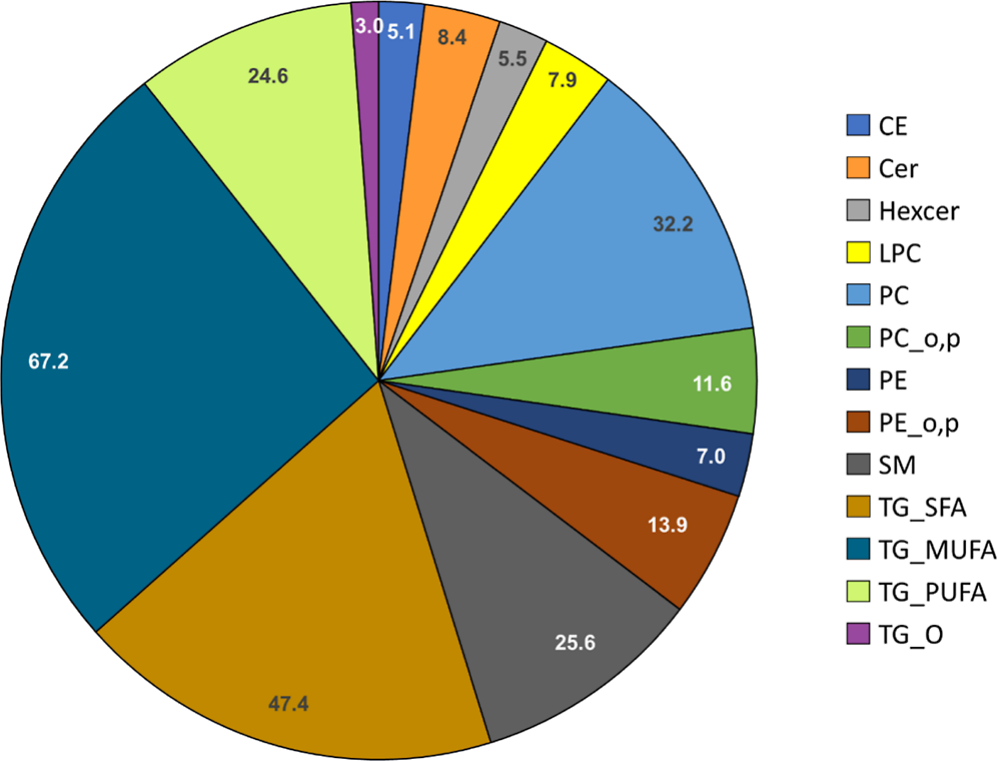
Composition (%) of lipids as lipid classes in breast milk samples.

248 lipids were identified from the breast milk (taken at delivery), with TGs being the most abundant group (Table S2, showing also RSD values and the level of identification based on Metabolomics Standard Initiative guidelines). The identified lipids included ceramides (Cer), hexosylceramides, dihexosylceramides, cholesterol esters (CE), TGs including ether-linked TGs (TG_O), diacylglycerols (DG), and phospholipids [lysophosphatidylcholines (LPC), phosphatidylcholines (PC), phosphatidylethanolamines (PE), phosphatidylinositols, and sphingomyelins (SM)] (Figure 1). Based on linear regression (adjusted with maternal age and BMI) PC, PE, and ether PEs were significantly different between the two groups (*p* < 0.05, logFC 0.31–0.36). Overall, the lipid composition in the HBM was similar to that reported in other studies.^23,47^

### Impact of Gestational Age, Maternal Age, and BMI, as Well as Other Maternal Lifestyle Factors on the Breast Milk Lipidome

**Figure 2:**
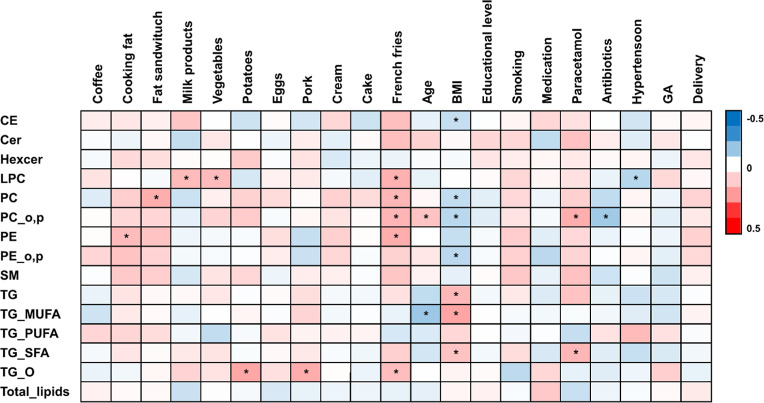
Partial correlations, adjusted by age and BMI (except for BMI and age) between maternal parameters and BM lipid classes. *p < 0.05.

We first investigated, using partial correlation, whether age, BMI, delivery type, gestational age, educational level, smoking, chronic diseases, medication, or dietary intake had an impact on the HBM lipidome, investigating both lipid classes as well as at the level of individual lipids (Figures 2 and S2 and Table S1), although none of the associations was strong (|*R*| < 0.2). Age and BMI both showed significant association with the HBM lipidome. Either gestational age or educational level did not show any significant associations at the lipid class level. Use of medication such as cortisone and paracetamol was associated with specific lipid classes; paracetamol showed positive association with saturated FA containing TGs, while use of cortisone showed inverse association with SM. Among the dietary factors, intake of milk products showed positive association with LPCs, type of cooking fat (level of saturated fat) showed positive association with alkylether PCs, vegetable intake showed adverse association with total TGs and PUFA-containing TGs, and intake of potatoes with TG_Os. Intake of fried potatoes, such as French fries, showed positive association with LPCs and saturated FA (SFA)-containing TGs and negative association with monounsaturated FA (MUFA)-containing TGs. Lake fish showed positive association with LPCs and other type of fish with hexylceramides. Omega-3 intake (estimated from the total fish intake) showed inverse association with Cer and hexylceramides.

At the level of individual lipids, BMI was associated with CE, age was associated with SFA-containing TGs and multiple phospholipids, and intake of milk products showed positive association with multiple SFA-containing TGs (Table S3).

### PFAS Exposure Has an Impact on the Breast Milk Lipidome

**Figure 3:**
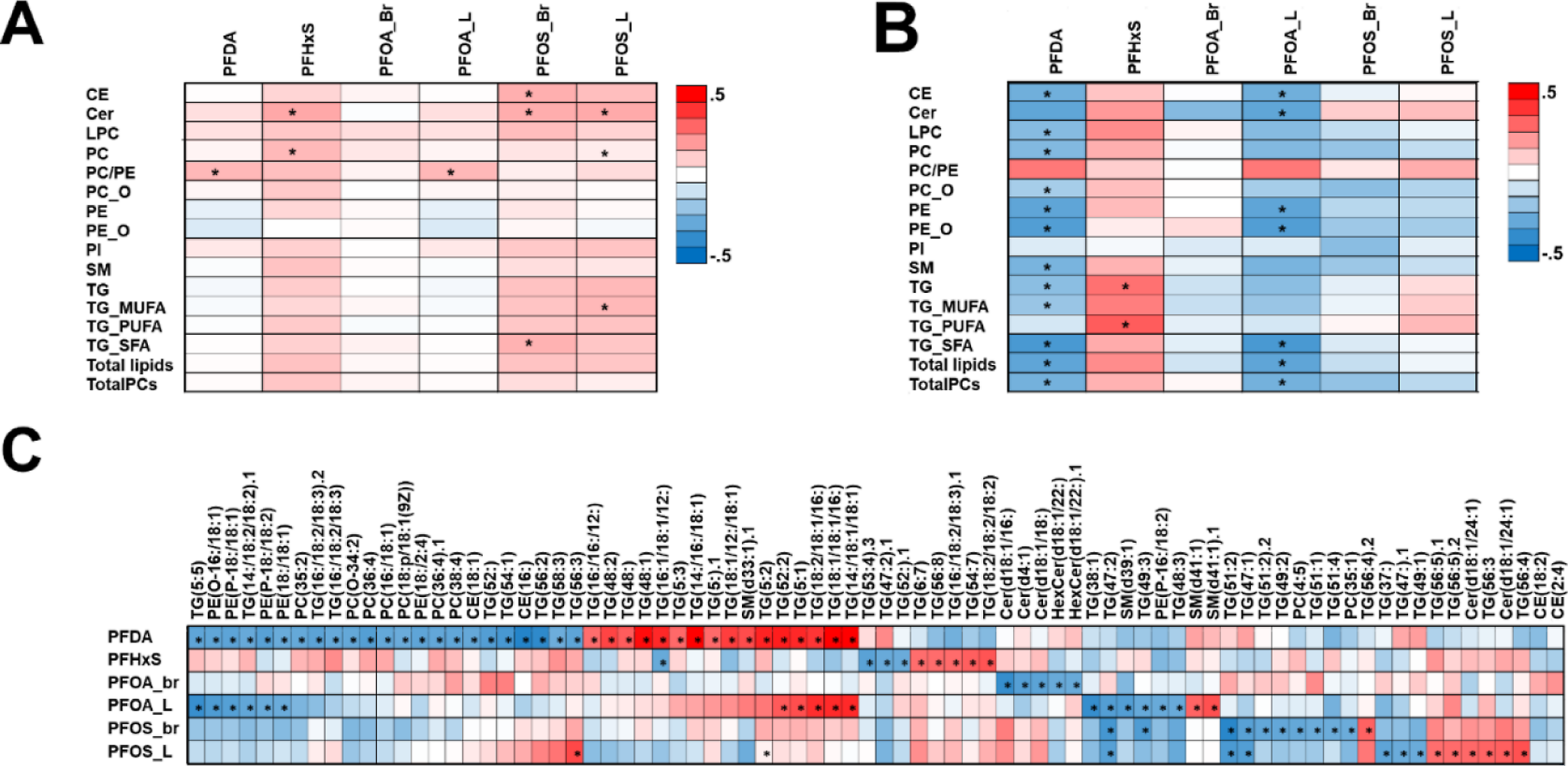
Partial correlation between circulating PFASs and (A) breast milk lipid classes in controls and (B) breast milk lipid classes in cases and (C) individual lipids in cases. Adjusted with age and BMI. *p < 0.01.

We conducted investigations into the impact of circulating PFAS on HBM lipid composition within the entire group as well as among those subjects whose children later developed autoimmune diseases. In the whole group, the PFASs were associated with increased levels of CE, Cer, PCs, and TGs containing SFA or MUFA (Figure 3A). The main drivers were PFHxS and branched and linear PFOS. Interestingly, when investigating the autoimmune group separately, we observed a different pattern, with PFAS levels associated with generally decreased HBM lipids, with PFDA and PFOA being the main drivers (Figure 3B). The PFHxS showed opposite but similar associations to the whole cohort, i.e., association with PFAS and increased TGs.

**Figure 4:**
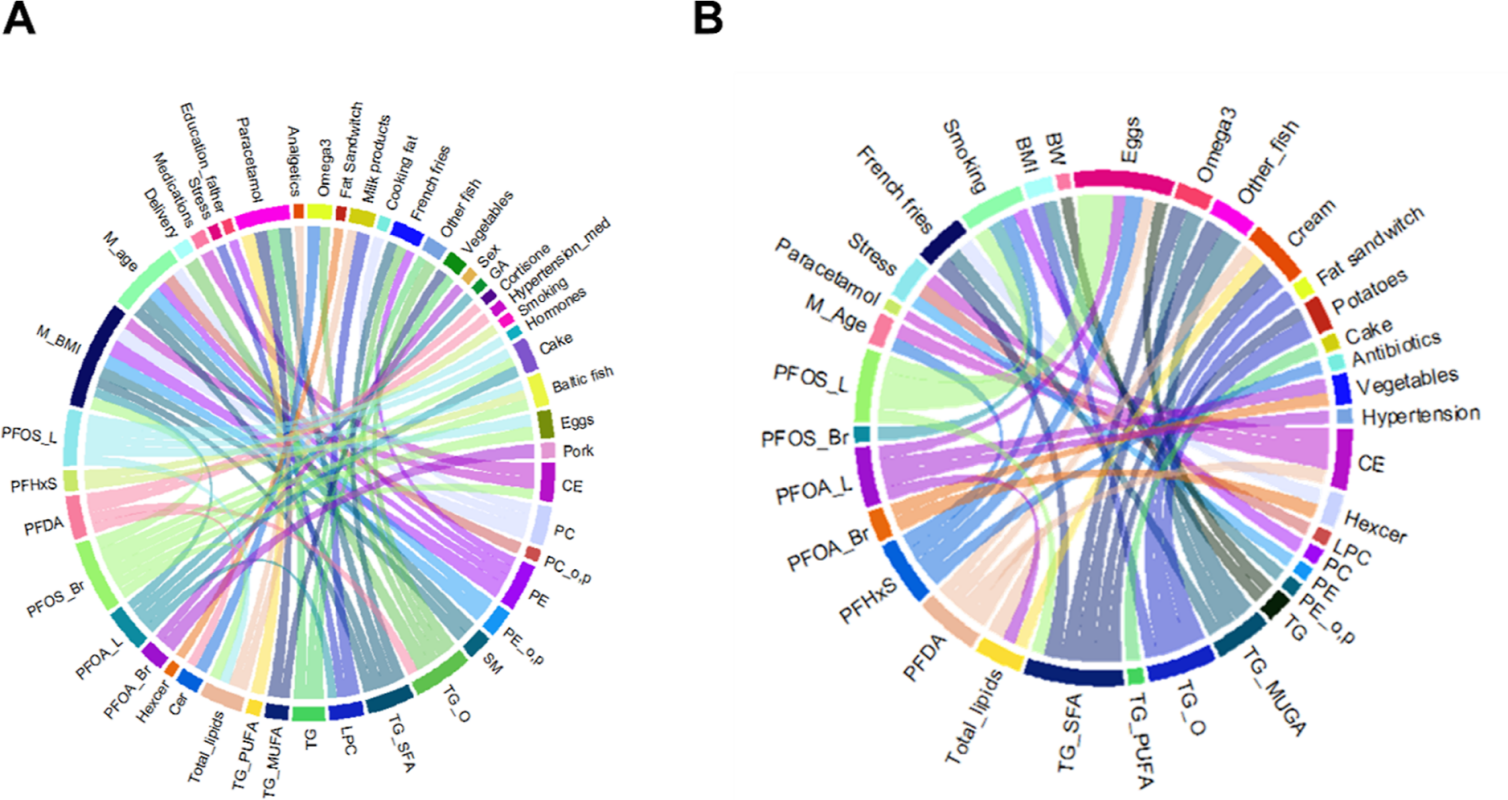
Chord plot of partial correlations between breast milk lipid classes, dietary parameters, lifestyle parameter, and other clinical data and circulating PFAS in (A) controls and (B) cases (*p* < 0.05), with intraclass correlations removed. Correlations adjusted with maternal age and BMI (except for maternal age and BMI).

On the level of individual lipids (Figure 3C and Table S4), several of the lipids were associated with circulating PFASs particularly in the case group, while the controls showed clearly weaker association with PFASs. The main driver of associations was PFDA, which showed positive association with SFA-containing TGs and negative association with PUFA-containing phospholipids. Linear PFOA showed similar associations while linear PFOS showed slightly different pattern, being positively associated with Cer and MUFA-containing TGs. In the control group, PFASs showed association mainly to lifestyle factors [diet, medication, and some clinical factors (maternal BMI, GA, and sex of the infant)], while in the case group, PFASs showed associations with all parameters, including HBM lipid classes (Figure 4). Overall, the external factors, especially PFASs, demonstrated a higher number of associations with the HBM lipidome in the case group compared to the control group, suggesting a greater overall impact due to the exposure.

While there are multiple studies reporting negative association with the total duration of breastfeeding and PFAS,^31^ we did not observe any significant association between PFASs and the duration of the breastfeeding, neither the duration of exclusive breastfeeding nor the total length. Between the two groups, the length of exclusive breastfeeding was slightly longer in the control group when compared with the group of future autoimmune disease (4.13 months vs 4.0 months, *p* = 0.033). However, in those studies reporting that PFASs are associated with shorter duration of total breastfeeding, the levels of PFOS and PFHxS have been significantly higher than in our study (PFOS 7.6–33.4 ng/mL and PFHxS 0.4–1.5 ng/mL).^31^

In this study, we observed that environmental factors had a marked impact on the HBM lipid composition. As reported in several epidemiological studies,^10,13,48,49^ including our recent study on Finnish cohort,^23^ our data in the current study also showed that the HBM lipid composition was affected by the age and BMI of the mothers, while maternal diet had comparatively weaker effect on the lipid composition, and education or smoking did not show significant associations. The variation of lipid composition of HBM has been attributed to cultural differences (including diet and other lifestyle factors) and genetic differences;^50^ however, most studies have investigated the associations on total lipid or lipid FA level only. Genetics was not included in the current study, and the cohort was from the geographically same area. In our study, the main finding was that the PFAS exposure was associated with reduced nutritional quality of the HBM, and importantly, this impact was significantly more pronounced in those mothers whose offspring later developed autoimmune/inflammatory diseases. This could imply interplay between environmental exposure and genetic factors or that reduced nutritional quality increases the risk of specific immune-mediated diseases.

When considering the whole study group, the PFAS exposure was associated with increased levels of Cer and CE and TGs containing either saturated or monounsaturated TGs, i.e., lipids that are generally considered to have poorer nutritional value. In the group of future autoimmune cases, we observed, in line with our previous study,^23^ that PFAS exposure was associated with overall reduction of the total lipids, as well as total phospholipids, and increased levels of SFA-containing TGs. Moreover, we observed that in this group, external factors, including diet, in addition to exposure, had more pronounced associations with the lipid profile in the HBM.

The mechanism how the PFAS may affect the HBM lipid composition is currently poorly understood. Data from both mouse models and humans suggest that PFAS exposure affects breast tissue development and that the exposure has an impact on lactation.^28,51^ It has been suggested that exposure to the PFAS can interrupt mammary gland development, as well as breast differentiation during late pregnancy and early lactation, and also change normal prolactin-family hormone secretion, which in turn is potentially linked with the detected reduction in breastfeeding duration due to PFAS exposure.^51^ In circulation, PFAS exposure has been associated with changes in metabolome,^52^ and particularly in the lipid profiles with changes reported in glycerophospholipid, linoleate/linoleic acid, sphingolipid, bile, and FA metabolism. This can potentially also be linked to the changes in the HBM composition.

The changes in lipid composition due to environmental factors, as shown in our study, are highly relevant for infant growth and development. For example, high content of choline-containing phospholipids (PC and SM) is important for organ growth and membrane biosynthesis since ca. 17% the neonate’s total choline intake is derived from these specific polar lipids.^9^

In our previous pilot study in a high T1D risk cohort, we observed a decreasing trend of PC/PE ratio with high PFAS exposure,^23^ indicating changes in the milk fat globule (MFG) size as these polar lipids are mainly located on the structured membrane and with the PC/PE ratio reflecting the size of the MFGs, with a high PC/PE ratio favoring small MFGs.^53^ In the current study, we observed the opposite trend in the control group, with a similar but not significant trend in the case group. The PFAS levels in the two studies were similar; therefore, this could not explain the different results obtained in the two studies. This could again implicate the role of genetic factors in the observed changes.

As a limitation of the study, the number of subjects within each disease group was low, and thus, we combined the groups together. This is an intrinsic limitation of the general population study setting when investigating diseases with low incidence. We also did not have information on the materials used for sample collection and storage, and the lipid quantitation was done with lipid-class specific calibration, and thus, the results are not fully quantitative. While the study cohort is representative of Swedish and Nordic population in general, the results may not be fully generalizable to overall population due to differences in dietary habits, socioeconomical factors, and ethnicity. However, within this study setting, a notable strength lies in its inclusivity beyond populations with a high genetic predisposition to certain diseases. Overall, it is challenging to establish robust cause-and-effect relationships between external and internal factors and HBM composition due to the dynamic nature of breast milk.^54^

In conclusion, our study suggests that breast milk lipid composition depends on a complex interaction between genetic and environmental factors, and importantly, this impact seems to be more pronounced in those mothers whose offspring later develop autoimmune/inflammatory diseases, even when there are not any major differences in the exposure levels. Hence, our results emphasize that solely measuring the PFAS concentration might not encompass the complete scope of the individual impacts of the chemical exposure. A comprehensive exposome approach is needed to evaluate the effects of PFAS exposure on HBM and, by extension, on the health outcomes of the offspring.

## Data Availability

Data from the clinical study are available upon request and an appropriate institutional collaboration agreement. These data are not available to access in a repository owing to concern that the identity of patients might be revealed inadvertently.

## Supporting Information Available

QC data (XLSX)
Maternal data from the ABIS cohort obtained through questionnaire; identified lipids with their mz values and retention times in breast milk with their RSD (%) in pooled QC samples and their level of identification; partial correlation of lipids with maternal BMI (adjusted with age), age (adjusted with BMI), and intake of milk products (adjusted with age and BMI); linear regression of breast milk lipids and serum PFAS concentrations, adjusted with age, BMI, delivery type, and gestational week; flowchart of the selection of the cohort; variation of lipids in breast milk, clinical variables, and lifestyle factors; partial correlations, adjusted with maternal BMI (except for BMI), between PFAS and maternal variables; and only those variables that show significant association with any of the PFAS (PDF)
