# Supplementary material for: Impact of Environmental Exposures on Human Breast Milk Lipidome in Future Immune-Mediated Diseases
Source: Environ Sci Technol. 2024 Jan 24;58(5):2214–23. doi: 10.1021/acs.est.3c06269 (PMC10851438; doi:10.1021/acs.est.3c06269)

Impact of environmental exposures on human breast milk lipidome in future immune-mediated diseases

Tuulia Hyötyläinen, Tannaz Ghaffarzadegan, Bagavathy Shanmugam Karthikeyan, Eric Triplett, Matej Orešič, Johnny Ludvigsson

Supplemental Material

Contents

Supplementary Table 1. Maternal data from ABIS cohort obtained through questionnaire. S2

Supplementary table 2. Identified lipids with their mz values and retention times in breast milk with their relative standard deviation (RSD%) in pooled QC samples and their level of identification. S3

Supplementary Table 3. Partial correlation of lipids with maternal BMI (adjusted with age), age (adjusted with BMI) and intake of milk products (adjusted with age and BMI). S10

Supplementary Table 4. Linear regression of breast milk lipids and serum PFAS concentrations, adjusted with age, BMI, delivery type and gestational week. S12

Supplementary Figure 1. Flowchart of the selection of the cohort. S15

Supplementary Figure 2. Variation of lipids in breast milk, clinical variables and lifestyle factors. S16

Supplementary Figure 3. Partial correlations, adjusted with maternal BMI (except for BMI), between PFAS and maternal variables. Only those variables that show significant association with any of the PFAS are shown. S17

Supplementary Table 1. Maternal data from ABIS cohort obtained through questionnaire.

**Diet questionary**	
How often did you drink coffee during the pregnancy?	Scale 1-5
What kind of cooking fat did you use?	Butter, margarine, butter-margarine mix, none (4, 3, 2,1, respectively)
What kind of fat did you use for sandwiches?	Butter, margarine, butter-margarine mix, none (4, 3, 2,1, respectively)
How much milk products did you drink or eat every day?	every day, 3-5 times a week, 1-2 times a week or less than 1-2 a week
How often did you eat vegetables during the pregnancy?	every day, 3-5 times a week, 1-2 times a week or less than 1-2 a week
How often did you eat potatoes during the pregnancy?	every day, 3-5 times a week, 1-2 times a week or less than 1-2 a week
How often did you eat eggs during the pregnancy?	every day, 3-5 times a week, 1-2 times a week or less than 1-2 a week
How often did you eat pork, bacon or sausages during the pregnancy?	every day, 3-5 times a week, 1-2 times a week or less than 1-2 a week
How often did you eat heavy cream or crème fraîche during the pregnancy?	every day, 3-5 times a week, 1-2 times a week or less than 1-2 a week
How often did you eat cakes, danish pastry or biscuits during the pregnancy?	every day, 3-5 times a week, 1-2 times a week or less than 1-2 a week
How often did you eat fried potatoes or french fries during the pregnancy?	every day, 3-5 times a week, 1-2 times a week or less than 1-2 a week
How often did you eat a) lake fish, b) Baltic fish or c) other type of fish	every day, 3-5 times a week, 1-2 times a week or less than 1-2 a week
How often did you eat omega-3 supplement?	Seldom, 1-2 a week, 3-5 times a week
**Health status**	**Procentige (%)**
T1D	1.4
T2D	0.5
Gestational diabetes	0.5
Lactose intolerance	1.8
Other food allergy	4.2
Celiac disease	0
**Medication**	**Procentige (%)**
Antibiotics	20.2
Cortisone	4.1
Hypertension medications	3.7
Psychotropics	0.9
Analgetics	51.8
Hormones	1.4
Chemotherapy	0.5
Other kind of medications	32.1
Paracetamol	17.4

Smoking	7.8

Supplementary table 2. Identified lipids with their mz values and retention times in breast milk with their relative standard deviation (RSD%) in pooled QC samples and their level of identification.

m/z	retention time	Lipid	RSD %	Level of Identification
369.3514	9.23	CE(16:0)	12.89	1
369.3514	8.82	CE(18:2)	30.24	1
369.3513	8.56	CE(16:1)	29.80	2
369.3514	9.26	CE(18:1)	29.93	1
604.602	7.68	Cer(d40:1)	15.32	2
520.5084	6.53	Cer(d18:1/16:0)	15.08	1
566.5508	6.96	Cer(d18:1/18:0)	13.65	1
622.6128	7.69	Cer(d18:1/22:0)	10.15	1
650.644	7.99	Cer(d18:1/24:0)	10.24	1
648.6284	7.61	Cer(d18:1/24:1)	6.41	1
632.6333	7.99	Cer(d42:1)	23.41	2
385.3459	5.66	Cholecalciferol (D3)	18.94	1
636.5554	6.76	DG (36:3)	33.60	1
784.6646	7.40	HexCer(d18:1/22:0)	10.94	1
812.6977	7.73	HexCer(d18:1/24:0)	10.51	2
862.6244	6.02	LacCer(d18:1/16:0)	16.32	1
468.308	2.85	LPC(14:0)	24.46	1
496.3396	3.24	LPC(16:0)	4.49	1
482.3609	3.46	LPC(16:0e)	16.85	2
524.3709	3.71	LPC(18:0)	4.91	1
520.3385	2.96	LPC(18:2)	9.06	1
480.3083	3.37	LysoPE(18:1)	47.17	1
734.5694	6.52	PC(16:0/16:0)	1.40	1
760.5848	6.50	PC(16:0/18:1)	1.54	1
746.6064	6.77	PC(16:0e/18:1(9Z))	4.15	1

790.6335	7.32	PC(18:0/18:0)	11.54	1
772.6219	6.84	PC(18:0p/18:1(9Z))	49.47	1
720.5534	6.29	PC(31:0)	5.13	2
732.5529	6.04	PC(32:1)	3.81	2
730.5378	5.60	PC(32:2)	17.52	2
748.5861	6.74	PC(33:0)	17.95	2
746.5678	6.28	PC(33:1)	10.77	2
758.5692	6.10	PC(34:2)	3.56	2
756.5529	5.73	PC(34:3)	19.66	2
774.6009	6.72	PC(35:1)	9.87	2
772.5829	6.33	PC(35:2)	14.86	2
788.6163	6.92	PC(36:1)	3.49	2
786.6005	6.55	PC(36:2)	2.06	2
784.5841	6.13	PC(36:3)	2.90	2
782.5668	6.50	PC(36:4)	2.95	2
782.5689	5.97	PC(36:4)	5.66	2
782.5681	5.70	PC(36:4)	28.58	2
815.6346	6.91	PC(37:3)	13.51	2
816.6502	7.26	PC(38:1)	15.37	2
814.6315	6.91	PC(38:2)	37.35	2
812.6155	6.62	PC(38:3)	5.76	2
810.5996	6.41	PC(38:4)	4.88	2
808.5843	5.96	PC(38:5)	7.35	2
806.5687	5.78	PC(38:6)	26.49	2
832.6645	7.34	PC(39:0)	5.22	2
836.6127	6.38	PC(40:5)	11.90	2
834.6012	6.24	PC(40:6)	13.99	2
720.5894	6.81	PC(O-32:0)	7.74	2
718.5789	6.73	PC(O-32:1)	23.20	2
744.5896	6.40	PC(O-34:2)	6.91	2
759.6367	7.06	PC(O-34:3) NH4 adduct	4.33	2
770.6046	6.37	PC(O-36:3)	21.70	2
768.5879	6.26	PC(O-36:4)	30.31	2

766.5712	6.16	PC(O-36:5)	19.21	2
794.6038	6.24	PC(O-38:5)	23.89	2
706.538	6.04	PC(15:0/15:0)	3.64	1
718.5378	6.61	PE(16:0/18:1)	3.67	1
716.5225	6.22	PE(16:0/18:2)	9.08	2
740.5243	6.08	PE(16:0/20:4)	15.16	2
726.5439	6.50	PE(16:1e/20:3)	6.89	2
722.5109	5.96	PE(16:1e/20:5)	22.30	2
746.5691	7.02	PE(18:0/18:1)	2.96	1
768.5534	6.52	PE(18:0/20:4)	7.19	2
792.5559	6.33	PE(18:0/22:6)	6.44	2
742.5372	6.23	PE(18:1/18:2)	22.50	2
780.5902	6.98	PE(18:1e/22:4)	15.30	2
776.5582	6.51	PE(18:1e/22:6)	17.80	2
702.5419	6.82	PE(O-16:0/18:1)	5.58	2
752.5581	6.71	PE(O-38:5) or PE(P-38:4)	5.72	2
752.5579	6.61	PE(O-38:5) or PE(P-38:4)	7.22	2
700.527	6.44	PE(P-16:0/18:2)	6.14	2
748.527	6.09	PE(P-16:0/22:6)	7.67	2
730.5738	7.19	PE(P-18:0/18:1)	5.87	2
728.5584	6.85	PE(P-18:0/18:2)	7.57	2
778.5742	6.67	PE(P-18:0/22:5) + PE(P-20:1/20:4)	26.16	2
904.5902	6.00	PI(18:0/20:4)	8.17	1
876.5692	6.54	PS(41:4)	14.25	2
807.6357	7.03	SM (d18:1/24:4)	10.56	2
835.666	7.37	SM (d18:1/26:4)	8.80	2
773.652	7.27	SM(d39:1)	15.18	2
811.6677	6.99	SM(d42:3)	4.23	2
745.6203	6.86	SM(37:1)	9.30	2
785.6518	7.04	SM(40:2)	4.06	2
701.5583	5.60	SM(d16:1/18:1) or SM(d18:2/16:0)	7.96	2
677.5569	5.75	SM(d18:0/14:0)	30.34	1
705.5862	6.28	SM(d18:0/16:0)	7.45	1

647.5099	4.98	SM(d18:1/12:0)	26.51	1
703.5743	6.13	SM(d18:1/16:0)	2.16	1
815.6993	7.81	SM(d18:1/24:0)	6.30	1
675.5425	5.57	SM(d32:1)	6.77	2
689.5583	5.86	SM(d33:1)	7.99	2
689.5582	5.91	SM(d33:1)	13.00	2
733.6163	6.74	SM(d36:0)	54.77	2
731.6056	6.63	SM(d36:1)	3.42	2
729.5897	6.14	SM(d36:2)	4.84	2
757.6209	6.62	SM(d38:2)	3.31	2
787.6682	7.46	SM(d40:1)	5.26	2
801.6836	7.64	SM(d41:1)	7.06	2
801.6834	7.70	SM(d41:1)	12.13	2
799.668	7.21	SM(d41:2)	6.71	2
813.6843	7.38	SM(d42:2)	5.11	2
822.7548	8.73	TG(14:0/16:0/18:1)	6.96	2
848.7705	8.68	TG(14:0/18:1/18:1)	6.30	2
844.7388	8.29	TG(14:0/18:2/18:2)	5.13	2
844.7393	8.15	TG(14:0/18:2/18:2)	5.14	2
768.7076	8.50	TG(16:0/16:0/12:0)	5.82	2
824.77	9.11	TG(16:0/16:0/16:0)	5.30	1
878.8156	9.39	TG(16:0/18:0/18:1)	4.24	2
792.7076	8.18	TG(16:0/18:2/12:0)	6.27	2
872.7702	8.53	TG(16:0/18:2/18:2)	5.35	2
872.7706	8.37	TG(16:0/18:2/18:2)	6.39	2
870.7545	8.15	TG(16:0/18:2/18:3)	4.81	2
870.7544	8.24	TG(16:0/18:2/18:3)	9.96	2
924.8016	8.42	TG(16:0/22:5/18:1) or TG(20:4/18:1/18:1)	7.40	2
924.8014	8.52	TG(16:0/22:5/18:1) or TG(20:4/18:1/18:1)	27.43	2
794.7235	8.46	TG(16:1/18:1/12:0)	6.30	2
908.8639	10.36	TG(18:0/18:0/18:0)	17.20	1
820.739	8.42	TG(18:1/12:0/18:1) or TG(18:2/16:0/14:0)	6.39	2
876.8008	8.97	TG(18:1/18:1/16:0)	7.13	2

902.8171	8.90	TG(18:1/18:1/18:1)	6.84	1
948.8013	8.29	TG(18:1/18:1/22:6)	5.40	2
948.8005	8.18	TG(18:1/18:1/22:6)	10.14	2
898.7857	8.48	TG(18:1/18:2/18:2)	5.40	2
898.786	8.34	TG(18:1/18:2/18:2)	5.43	2
874.7861	8.64	TG(18:2/18:1/16:0)	6.39	2
900.8018	8.59	TG(18:2/18:1/18:1)	6.05	2
900.8013	8.81	TG(18:2/18:1/18:1)	6.29	2
896.7702	8.11	TG(18:2/18:2/18:2) or TG(18:3/18:2/18:1)	3.75	2
896.7706	8.27	TG(18:2/18:2/18:2) or TG(18:3/18:2/18:1)	5.71	2
922.7859	8.32	TG(18:2/22:5/16:0)	6.17	2
922.7853	8.19	TG(18:2/22:5/16:0)	8.68	2
670.599	7.58	TG(37:0)	12.35	2
682.5976	7.45	TG(38:1)	50.23	2
796.7388	8.79	TG(46:0)	5.72	2
810.7539	8.88	TG(47:0)	6.69	2
808.7389	8.58	TG(47:1)	5.55	2
806.7229	8.29	TG(47:2)	5.10	2
806.7227	8.53	TG(47:2)	20.23	2
829.7253	9.11	TG(48:0)	9.76	2
827.7098	8.73	TG(48:1)	12.20	2
825.6941	8.42	TG(48:2)	10.90	2
818.7233	8.14	TG(48:3)	6.01	2
838.7856	9.22	TG(49:0)	5.30	2
838.7854	9.28	TG(49:0)	6.40	2
836.7706	8.87	TG(49:1)	4.92	2
834.7546	8.55	TG(49:2)	5.74	2
832.7384	8.27	TG(49:3)	7.05	2
852.801	9.48	TG(50:0)	3.42	2
857.7583	8.64	TG(50:0)	16.47	2
850.7858	9.04	TG(50:1)	6.18	2
855.7426	8.37	TG(50:1)	18.51	2
853.7255	8.69	TG(50:2)	12.69	2

846.7548	8.38	TG(50:3)	7.07	2
851.7098	8.38	TG(50:3)	10.28	2
842.7224	7.91	TG(50:5)	4.87	2
842.7228	8.00	TG(50:5)	18.63	2
864.8017	9.21	TG(51:1)	4.44	2
862.7855	9.19	TG(51:2)	4.23	2
862.786	8.82	TG(51:2)	5.17	2
860.7701	8.52	TG(51:3)	5.86	2
860.7702	8.85	TG(51:3)	14.17	2
858.754	8.28	TG(51:4)	5.40	2
858.7502	8.01	TG(51:4)	14.16	2
880.8328	9.89	TG(52:0)	2.91	2
880.8244	9.38	TG(52:0)	11.01	2
881.7568	8.97	TG(52:2)	12.43	2
879.7411	8.64	TG(52:3)	12.14	2
877.7255	8.39	TG(52:4)	12.72	2
877.7251	8.53	TG(52:4)	13.23	2
875.7093	8.23	TG(52:5)	10.68	2
875.7092	8.15	TG(52:5)	11.56	2
868.7385	8.08	TG(52:6)	4.45	2
868.7384	7.94	TG(52:6)	6.35	2
868.739	8.12	TG(52:6)	6.45	2
890.8171	9.13	TG(53:2)	5.67	2
888.8013	8.77	TG(53:3)	5.72	2
886.7853	8.52	TG(53:4)	4.65	2
886.7776	8.26	TG(53:4)	12.83	2
886.7879	8.76	TG(53:4)	19.50	2
884.7687	8.28	TG(53:5)	35.13	2
906.8482	9.77	TG(54:1)	3.87	2
909.7883	9.29	TG(54:2)	11.89	2
907.7723	8.90	TG(54:3)	11.42	2
905.7567	8.59	TG(54:4)	11.90	2
905.7564	8.81	TG(54:4)	12.87	2

903.7405	8.34	TG(54:5)	11.56	2
903.7409	8.48	TG(54:5)	13.46	2
901.7255	8.35	TG(54:6)	13.22	2
901.7251	8.26	TG(54:6)	14.17	2
901.7244	8.11	TG(54:6)	17.31	2
894.7548	8.09	TG(54:7)	6.81	2
894.7534	7.94	TG(54:7)	14.35	2
920.8578	9.42	TG(55:1)	47.59	2
912.8003	8.54	TG(55:5)	20.02	2
932.8642	9.64	TG(56:2)	2.80	2
930.8486	9.20	TG(56:3)	4.39	2
928.8331	8.84	TG(56:4)	5.99	2
928.8321	9.12	TG(56:4)	7.02	2
928.8322	8.98	TG(56:4)	7.33	2
933.7877	8.84	TG(56:4)	13.37	2
926.8167	8.57	TG(56:5)	5.71	2
926.8168	8.75	TG(56:5)	10.61	2
929.7563	8.43	TG(56:6)	16.21	2
920.7703	8.08	TG(56:8)	6.19	2
918.7548	7.88	TG(56:9)	4.69	2
944.7704	7.85	TG(58:10)	10.08	2
958.8797	9.54	TG(58:3)	3.45	2
952.8321	8.80	TG(58:6)	6.58	2
952.8321	8.86	TG(58:6)	9.17	2
952.8308	8.58	TG(58:6)	13.99	2
946.7856	8.06	TG(58:9)	5.51	2
978.8471	8.56	TG(60:7)	8.24	2
978.8476	8.82	TG(60:7)	10.43	2
836.8059	9.58	TG(O-50:1) or TG(P-50:0)	11.79	2
864.8371	10.01	TG(O-52:1) or TG(P-52:0)	14.15	2
862.8216	9.48	TG(O-52:2) or TG(P-52:1)	11.37	2

Supplementary Table 3. Partial correlation of lipids with maternal BMI (adjusted with age), age (adjusted with BMI) and intake of milk products (adjusted with age and BMI).

**Age**	**R**	**p-value**	**FDR**
PC(35:1)	0.31	**2.69E-06**	**0.001**
PC(32:2)	0.25	**1.89E-04**	**0.021**
PC/15:0/15:0)	0.25	**2.43E-04**	**0.021**
SM (d39:1)	0.23	**6.16E-04**	**0.04**
TG(16:0/16:0/16:0)	0.22	**8.14E-04**	**0.04**
SM(d32:1)	0.22	**9.40E-04**	**0.04**
PC(33:1)	0.22	**1.05E-03**	**0.04**
SM(37:1)	0.21	**1.74E-03**	**0.057**
TG(16:0/18:2/18:2)	-0.21	**2.18E-03**	**0.064**
TG(50:1).1	-0.2	**2.54E-03**	**0.067**
TG(47:0).1	0.2	**3.62E-03**	**0.083**
TG(48:0)	0.19	**4.12E-03**	**0.083**
TG(46:0)	0.19	**4.15E-03**	**0.083**
PC(35:2)	0.19	**4.41E-03**	**0.083**
PC(38:5)	0.19	**5.88E-03**	**0.093**
SM(d33:1)	0.18	**6.04E-03**	**0.093**
TG(54:3)	-0.18	**6.50E-03**	**0.093**
SM(d18:0/16:0)	0.18	**7.07E-03**	**0.093**
Cer(d18:1/24:0)	0.18	**7.28E-03**	**0.093**

PC(O-36:4)	0.18	**7.39E-03**	**0.093**
TG(47:0)	0.18	**7.47E-03**	**0.093**
PE(16:0/18:2)	0.18	**7.84E-03**	**0.093**
SM(d36:1)	0.18	**9.35E-03**	**0.094**
TG(47:2).1	0.18	**9.42E-03**	**0.094**
TG(50:0)	0.17	**9.73E-03**	**0.094**
TG(56:3)	-0.17	**1.00E-02**	**0.094**
SM(d41:2)	0.17	**1.03E-02**	**0.094**
**BMI**	**R**	**p-value**	**FDR**
CE(16:0)	-0.23	**7.34E-04**	**0.007**
CE(18:1)	-0.13	**5.61E-02**	0.147
CE(18:2)	-0.11	**9.75E-02**	0.224
CE(20:4)	-0.16	**1.82E-02**	**0.07**
**Milk Products**	**R**	**p-value**	**FDR**
TG(47:0).1	0.28	**2.66E-05**	**0.007**
TG(47:0)	0.26	**1.30E-04**	**0.017**
TG(52:4).1	0.24	**3.53E-04**	**0.026**
TG(48:0)	0.24	**3.91E-04**	**0.026**
TG(49:1)	0.23	**6.37E-04**	**0.034**
TG(51:2)	0.22	**8.32E-04**	**0.037**
TG(16:0/16:0/16:0)	0.21	**1.46E-03**	**0.055**

Supplementary Table 4. Linear regression of breast milk lipids and serum PFAS concentrations, adjusted with age, BMI, delivery type and gestational week.

	**PFDA**			**PFHxS**			**PFOA_br**			**PFOA_L**			**PFOS_br**			**PFOS_L**		
**Lipid**	**t**	**p**	**Adj. p**	**t**	**p**	**adj.p**	**t**	**p**	**adj.p**	**t**	**p**	**adj.p**	**t**	**pe**	**adj.p**	**t**	**p**	**adj.p**
TG(52:2)	3.48	**0.0005**	**0.07**	1.59	0.1128	0.61	-0.45	0.6542	0.93	2.61	**0.0090**	0.18	1.60	0.1107	0.48	0.27	0.7876	0.96
TG(50:2)	3.35	**0.0008**	**0.07**	1.59	0.1109	0.61	-0.51	0.6132	0.93	2.43	**0.0152**	0.20	1.54	0.1248	0.49	0.16	0.8691	0.97
CE fragment	-3.32	**0.0009**	**0.07**	-1.00	0.3184	0.81	0.16	0.8743	0.96	-1.61	0.1084	0.50	-1.00	0.3193	0.69	0.18	0.8582	0.97
TG(18:1/18:1/16:0)	3.17	**0.0016**	**0.10**	1.98	**0.0472**	0.52	-0.53	0.5941	0.93	3.19	**0.0014**	**0.10**	2.00	**0.0455**	0.39	1.03	0.3023	0.73
TG(14:0/18:1/18:1)	2.97	**0.0030**	0.13	2.05	**0.0403**	0.52	-0.78	0.4352	0.93	3.25	**0.0012**	**0.10**	2.02	**0.0439**	0.39	1.09	0.2746	0.72
TG(48:1)	2.96	**0.0031**	0.13	0.48	0.6310	0.87	-0.78	0.4366	0.93	0.91	0.3636	0.74	0.46	0.6462	0.85	-0.67	0.5013	0.86
TG(50:0).1	2.80	**0.0051**	0.18	1.74	0.0817	0.60	-0.50	0.6155	0.93	2.14	**0.0323**	0.24	1.59	0.1119	0.48	0.43	0.6682	0.94
TG(14:0/16:0/18:1)	2.74	**0.0062**	0.19	0.96	0.3396	0.82	-0.74	0.4586	0.93	1.75	0.0797	0.43	0.98	0.3253	0.69	0.09	0.9270	0.99
TG(52:3)	2.66	**0.0077**	0.21	1.49	0.1366	0.61	-0.17	0.8682	0.96	2.26	**0.0236**	0.22	1.41	0.1573	0.59	0.26	0.7963	0.96
TG(18:2/18:1/16:0)	2.49	**0.0127**	0.32	2.00	**0.0456**	0.52	-0.37	0.7103	0.95	3.14	**0.0017**	**0.10**	1.99	**0.0468**	0.39	1.28	0.2018	0.71
PC(38:4)	-2.44	**0.0146**	0.33	-1.61	0.1082	0.61	-0.02	0.9866	0.99	-1.55	0.1202	0.51	-1.74	0.0816	0.43	-0.62	0.5324	0.87
TG(50:1)	2.41	**0.0161**	0.33	1.53	0.1259	0.61	-0.49	0.6230	0.93	2.76	**0.0057**	0.15	1.66	0.0970	0.46	0.74	0.4595	0.86
PC(36:4)	-2.34	**0.0193**	0.37	-1.08	0.2790	0.76	-0.47	0.6361	0.93	-1.10	0.2716	0.69	-1.57	0.1163	0.48	-0.99	0.3217	0.74

TG(16:1/18:1/12:0)	2.28	**0.0226**	0.37	0.46	0.6433	0.87	-0.65	0.5189	0.93	0.70	0.4837	0.79	0.39	0.6937	0.85	-0.39	0.6961	0.94
TG(48:2)	2.25	**0.0243**	0.37	0.77	0.4429	0.85	-0.60	0.5479	0.93	0.70	0.4835	0.79	0.53	0.5989	0.84	-0.37	0.7144	0.95
PC(16:0/18:1)	-2.25	**0.0244**	0.37	-1.01	0.3129	0.81	-0.67	0.5020	0.93	-0.99	0.3212	0.74	-1.35	0.1784	0.60	-0.81	0.4159	0.85
PC(36:4).1	-2.24	**0.0251**	0.37	-0.75	0.4514	0.85	-0.56	0.5743	0.93	-0.57	0.5703	0.86	-0.84	0.4031	0.75	-0.08	0.9326	0.99
TG(50:3).1	2.13	**0.0335**	0.39	1.29	0.1955	0.68	-0.58	0.5642	0.93	1.19	0.2332	0.66	0.79	0.4268	0.76	0.01	0.9957	1.00
SM(d41:1).2	-2.09	**0.0368**	0.39	0.92	0.3555	0.82	-0.46	0.6433	0.93	0.09	0.9277	0.97	0.40	0.6861	0.85	0.51	0.6117	0.91
SM(d38:2)	-2.07	**0.0383**	0.39	-0.55	0.5842	0.87	-0.91	0.3609	0.93	-1.09	0.2778	0.70	-1.10	0.2692	0.69	-0.55	0.5802	0.89
TG(52:0)	-2.05	**0.0400**	0.39	-3.34	**0.0008**	**0.10**	0.59	0.5535	0.93	-2.35	**0.0187**	0.21	-3.01	**0.0026**	0.16	-2.65	**0.0081**	0.67
TG(50:5)	-2.05	**0.0401**	0.39	-0.38	0.7029	0.87	0.52	0.6055	0.93	-1.95	0.0507	0.33	-0.71	0.4776	0.77	0.23	0.8162	0.96
TG(18:0/18:0/18:0)	-2.05	**0.0402**	0.39	-1.43	0.1520	0.62	0.36	0.7189	0.95	-2.23	**0.0261**	0.22	-1.36	0.1724	0.60	-1.01	0.3146	0.74
PC(34:2)	-2.03	**0.0429**	0.39	-1.26	0.2067	0.68	-0.89	0.3722	0.93	-1.20	0.2293	0.66	-1.58	0.1142	0.48	-0.95	0.3441	0.76
SM(d33:1).1	2.01	**0.0443**	0.39	-0.90	0.3667	0.83	0.38	0.7048	0.95	0.83	0.4082	0.74	-1.05	0.2954	0.69	-1.18	0.2363	0.71
TG(18:1/12:0/18:1)	2.01	**0.0446**	0.39	1.28	0.1994	0.68	-0.66	0.5114	0.93	1.77	0.0772	0.43	1.15	0.2504	0.68	0.62	0.5355	0.87
PC(38:5)	-2.00	**0.0456**	0.39	-0.95	0.3443	0.82	-0.07	0.9405	0.98	-0.22	0.8284	0.93	-0.88	0.3794	0.71	-0.13	0.8976	0.99
TG(56:9)	-1.97	**0.0483**	0.39	0.08	0.9327	0.97	0.73	0.4665	0.93	-0.34	0.7338	0.92	0.05	0.9614	0.97	1.04	0.2998	0.73
TG(54:2).1	1.97	**0.0484**	0.39	-0.69	0.4930	0.85	0.20	0.8407	0.96	0.38	0.7055	0.92	-0.41	0.6829	0.85	-1.04	0.2966	0.73
TG(54:1)	-1.97	**0.0484**	0.39	-2.55	**0.0107**	0.42	0.69	0.4928	0.93	-2.26	**0.0239**	0.22	-2.31	**0.0208**	0.30	-2.02	**0.0430**	0.67
TG(56:2)	-1.97	**0.0487**	0.39	-1.30	0.1946	0.68	0.97	0.3307	0.93	-1.56	0.1182	0.51	-1.05	0.2949	0.69	-0.68	0.4980	0.86
PC(40:5)	-1.57	0.1173	0.46	-3.68	**0.0002**	**0.06**	-0.84	0.4027	0.93	-2.83	**0.0047**	0.15	-3.91	**0.0001**	**0.02**	-2.79	**0.0053**	0.67
PE(16:1e/20:3).1	-0.91	0.3618	0.66	-2.87	**0.0041**	0.33	-0.58	0.5591	0.93	-2.79	**0.0053**	0.15	-3.16	**0.0016**	0.16	-2.15	**0.0314**	0.67
PE(18:0/18:1)	-1.57	0.1163	0.46	-2.76	**0.0057**	0.35	-0.74	0.4600	0.93	-3.21	**0.0013**	**0.10**	-3.10	**0.0019**	0.16	-2.35	**0.0188**	0.67
PE(O-38:5)	-1.68	0.0938	0.46	-2.53	**0.0114**	0.42	-0.50	0.6196	0.93	-2.20	**0.0281**	0.22	-2.77	**0.0056**	0.20	-1.52	0.1295	0.69
PE(P-18:0/18:1)	-1.47	0.1422	0.47	-2.48	**0.0133**	0.42	-1.39	0.1641	0.93	-3.04	**0.0024**	0.12	-2.85	**0.0044**	0.20	-2.20	**0.0281**	0.67
PC(38:3)	-1.84	0.0665	0.46	-2.39	**0.0169**	0.42	0.10	0.9208	0.98	-2.73	**0.0064**	0.15	-2.39	**0.0167**	0.30	-1.86	0.0635	0.67
CE(18:2)	-0.48	0.6331	0.82	-2.37	**0.0179**	0.42	0.36	0.7224	0.95	-2.09	**0.0369**	0.26	-2.57	**0.0101**	0.29	-2.28	**0.0227**	0.67
PS(41:4)	-1.55	0.1220	0.46	-2.35	**0.0190**	0.42	0.04	0.9653	0.99	-2.39	**0.0169**	0.20	-2.76	**0.0057**	0.20	-2.27	**0.0235**	0.67
TG(54:1).1	0.59	0.5581	0.80	-2.28	**0.0226**	0.42	-0.30	0.7669	0.96	-1.55	0.1205	0.51	-2.15	**0.0313**	0.33	-2.55	**0.0107**	0.67
PC(36:2)	-1.72	0.0862	0.46	-2.25	**0.0242**	0.42	-0.08	0.9378	0.98	-2.39	**0.0167**	0.20	-2.56	**0.0104**	0.29	-1.86	0.0635	0.67
PE(18:0/20:4)	-1.67	0.0948	0.46	-2.24	**0.0251**	0.42	-0.69	0.4885	0.93	-2.22	**0.0263**	0.22	-2.32	**0.0202**	0.30	-1.49	0.1367	0.69
PE(18:0/22:6)	-1.04	0.2981	0.62	-2.23	**0.0259**	0.42	-0.36	0.7160	0.95	-2.25	**0.0244**	0.22	-2.37	**0.0179**	0.30	-1.79	0.0742	0.67
TG(50:0)	-1.74	0.0822	0.46	-2.22	**0.0266**	0.42	0.26	0.7914	0.96	-1.63	0.1041	0.49	-2.04	**0.0409**	0.38	-1.87	0.0610	0.67
LysoPE(18:1)	-1.16	0.2461	0.57	-2.21	**0.0270**	0.42	0.50	0.6180	0.93	-2.52	**0.0119**	0.20	-2.28	**0.0226**	0.31	-1.46	0.1456	0.69
TG(50:3)	1.64	0.1006	0.46	2.12	**0.0343**	0.50	-0.84	0.3983	0.93	2.42	**0.0157**	0.20	1.66	0.0976	0.46	1.41	0.1576	0.70
PE(18:1e/22:4)	-0.83	0.4046	0.70	-2.03	**0.0428**	0.52	-0.17	0.8633	0.96	-1.16	0.2481	0.67	-2.32	**0.0204**	0.30	-1.32	0.1869	0.71

PE(18:1e/22:6)	-0.37	0.7123	0.86	-1.96	**0.0496**	0.52	-0.35	0.7259	0.95	-1.38	0.1663	0.58	-2.21	**0.0272**	0.33	-1.51	0.1310	0.69
PC(35:2)	-1.60	0.1102	0.46	-1.96	**0.0497**	0.52	-0.28	0.7805	0.96	-2.86	**0.0043**	0.15	-2.32	**0.0203**	0.30	-1.74	0.0811	0.67
SM(d33:1)	0.43	0.6637	0.82	-1.03	0.3048	0.80	-2.24	**0.0251**	0.93	-0.38	0.7056	0.92	-1.10	0.2695	0.69	-1.20	0.2300	0.71
HexCer(d18:1/22:0)	0.45	0.6542	0.82	-0.62	0.5322	0.87	-1.98	**0.0478**	0.93	-0.24	0.8087	0.92	-0.93	0.3537	0.70	-1.19	0.2355	0.71
PC(32:2)	-0.56	0.5740	0.80	-0.28	0.7779	0.89	-1.97	**0.0492**	0.93	-0.93	0.3539	0.74	-0.38	0.7048	0.85	-0.08	0.9369	0.99
PE(P-18:0/18:2)	-1.29	0.1973	0.54	-1.94	0.0524	0.52	-0.91	0.3613	0.93	-2.72	**0.0066**	0.15	-2.49	**0.0127**	0.30	-1.92	0.0552	0.67
PE(16:0/18:1)	-1.51	0.1323	0.47	-1.92	0.0543	0.52	-0.74	0.4591	0.93	-2.60	**0.0093**	0.18	-2.35	**0.0187**	0.30	-1.79	0.0742	0.67
SM(d41:1).1	0.93	0.3522	0.65	1.21	0.2249	0.69	-0.93	0.3532	0.93	2.46	**0.0140**	0.20	0.98	0.3256	0.69	0.54	0.5909	0.90
PE(O-16:0/18:1)	-1.73	0.0842	0.46	-1.55	0.1223	0.61	-1.31	0.1914	0.93	-2.43	**0.0150**	0.20	-1.95	0.0508	0.41	-1.31	0.1895	0.71
TG(38:1)	-1.11	0.2654	0.57	-0.93	0.3515	0.82	-0.13	0.8949	0.96	-2.41	**0.0159**	0.20	-1.28	0.1991	0.63	-0.64	0.5205	0.86
TG(18:1/18:1/18:1)	0.13	0.8935	0.93	1.54	0.1242	0.61	0.71	0.4751	0.93	2.29	**0.0220**	0.22	1.85	0.0641	0.42	2.21	**0.0270**	0.67
PE(P-16:0/18:2)	-0.80	0.4209	0.72	-1.79	0.0734	0.59	-1.15	0.2506	0.93	-2.27	**0.0233**	0.22	-2.21	**0.0270**	0.33	-1.70	0.0896	0.68
PC(37:3)	-1.68	0.0931	0.46	-1.57	0.1162	0.61	-1.92	0.0550	0.93	-2.27	**0.0233**	0.22	-1.68	0.0929	0.45	-0.83	0.4055	0.85
TG(54:3)	0.95	0.3445	0.65	1.47	0.1418	0.62	0.70	0.4828	0.93	2.20	**0.0278**	0.22	1.73	0.0833	0.43	1.55	0.1218	0.69
PE(16:0/18:2)	-1.30	0.1939	0.54	-1.81	0.0700	0.59	-1.26	0.2062	0.93	-2.15	**0.0312**	0.23	-2.12	**0.0342**	0.33	-1.64	0.1009	0.69
PC(36:1)	-1.70	0.0897	0.46	-1.83	0.0680	0.59	-0.14	0.8915	0.96	-2.08	**0.0379**	0.26	-1.92	0.0554	0.42	-1.38	0.1691	0.70
PE(P-18:0/22:5) + PE(P-20:1/20:4)	-1.27	0.2024	0.54	-1.66	0.0976	0.61	-0.81	0.4163	0.93	-1.28	0.2010	0.63	-2.16	**0.0307**	0.33	-1.13	0.2581	0.71
PE(P-16:0/22:6)	-1.21	0.2279	0.55	-1.89	0.0588	0.54	-0.65	0.5142	0.93	-1.66	0.0972	0.48	-2.13	**0.0329**	0.33	-1.35	0.1772	0.71
TG(51:1)	-0.79	0.4320	0.72	-1.66	0.0978	0.61	0.22	0.8221	0.96	-1.25	0.2109	0.64	-2.13	**0.0333**	0.33	-2.02	**0.0435**	0.67
PC(35:1)	-1.43	0.1521	0.48	-1.64	0.1019	0.61	-0.81	0.4197	0.93	-1.54	0.1225	0.51	-2.12	**0.0343**	0.33	-1.98	**0.0481**	0.67
TG(18:2/18:1/18:1)	-0.51	0.6075	0.82	1.60	0.1100	0.61	0.91	0.3654	0.93	1.94	0.0522	0.33	1.87	0.0618	0.42	2.37	**0.0180**	0.67
TG(18:1/18:2/18:2).1	-1.15	0.2517	0.57	1.25	0.2103	0.68	0.90	0.3700	0.93	1.04	0.2997	0.74	1.44	0.1513	0.58	2.18	**0.0290**	0.67

Supplementary Figure 1. Flowchart of the selection of the cohort.

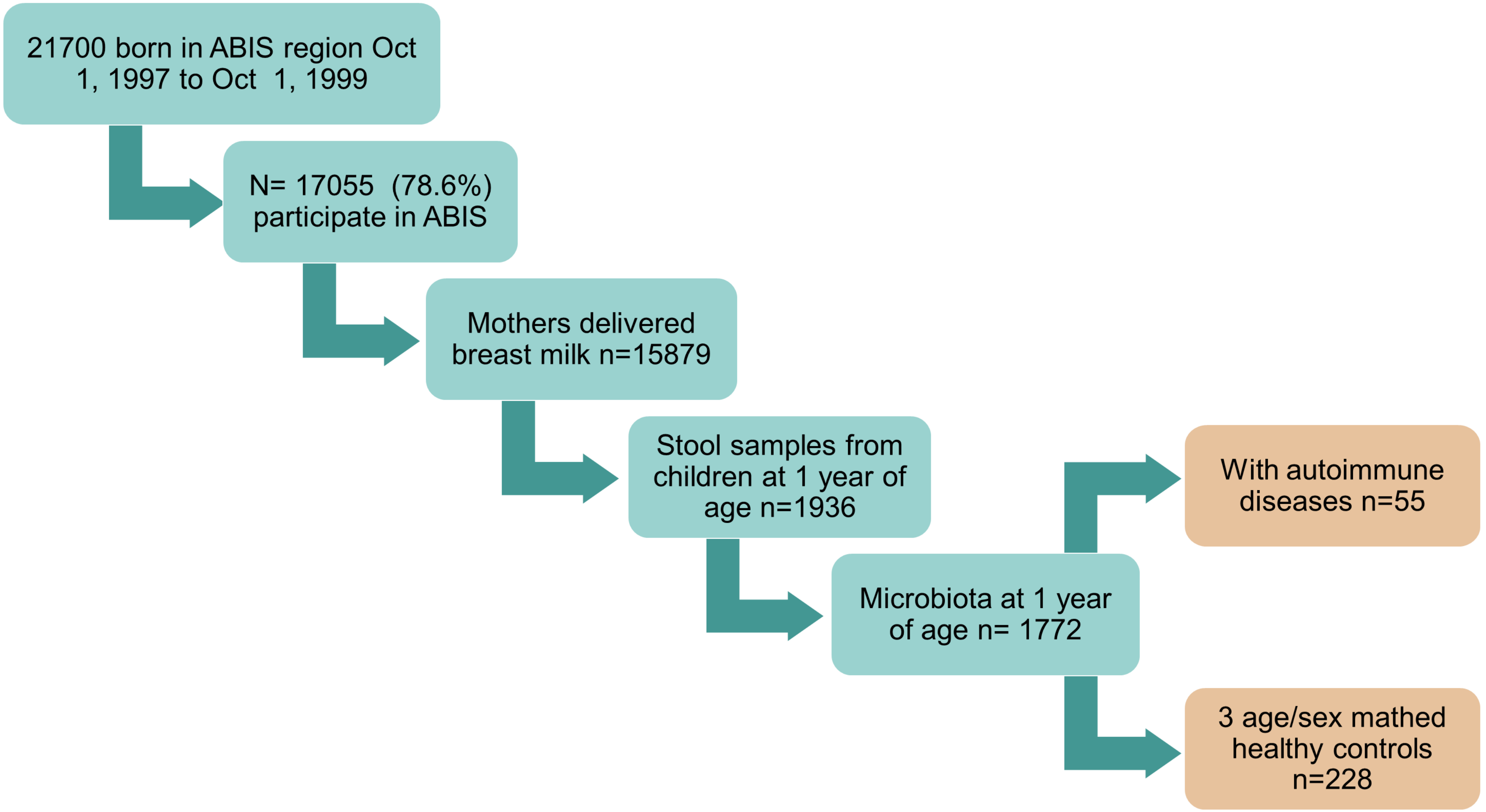

Supplementary Figure 2. Variation of lipids in breast milk, clinical variables and lifestyle factors.

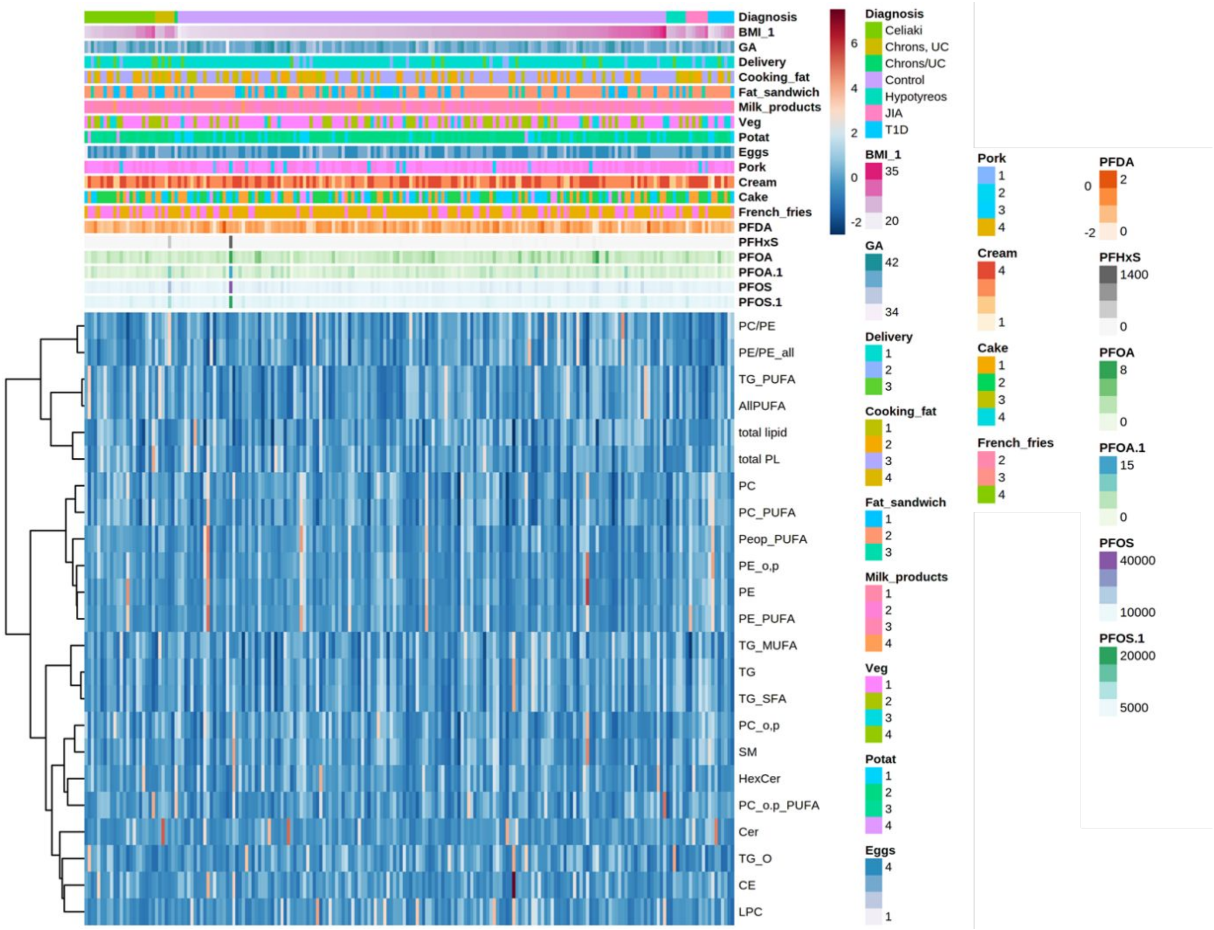

Supplementary Figure 3. Partial correlations, adjusted with maternal BMI (except for BMI), between PFAS and maternal variables. Only those variables that show significant association with any of the PFAS are shown.

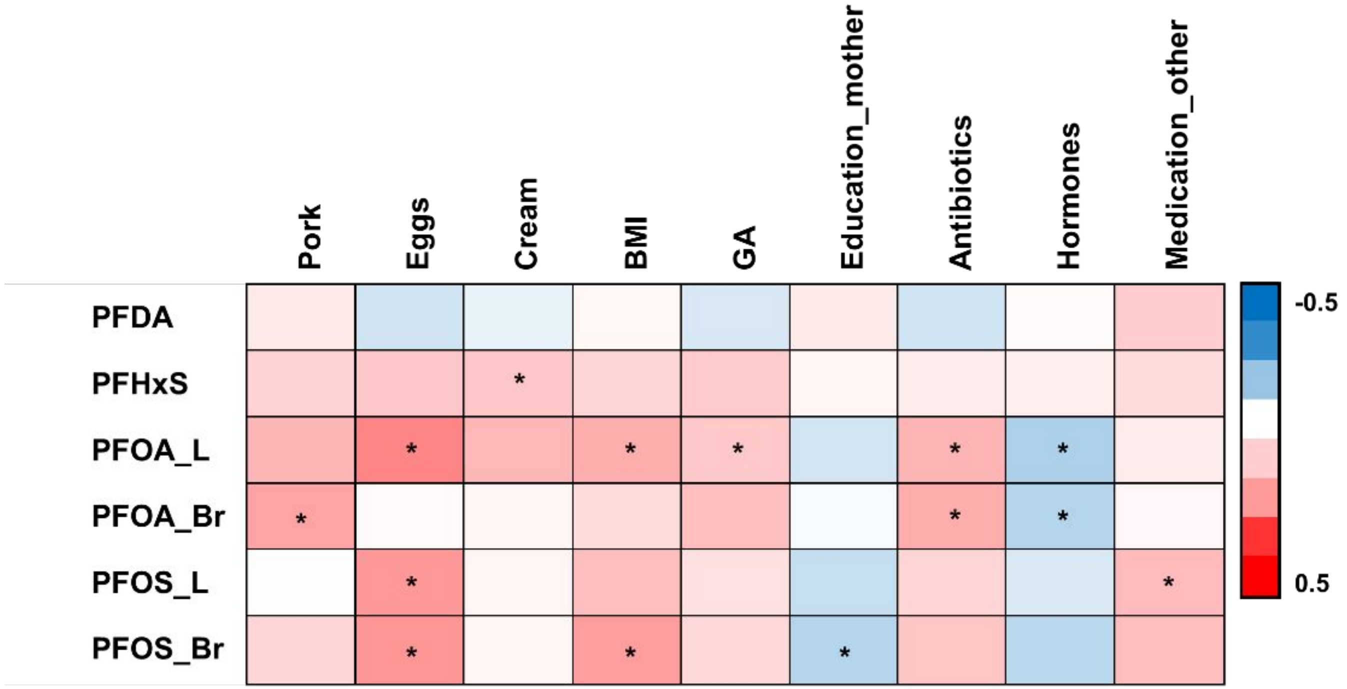

Supplement: Supplementary file 2 — es3c06269_si_002.pdf [file es3c06269_si_002.pdf]
